# Use of scenario ensembles for deriving seismic risk

**DOI:** 10.1073/pnas.1807433115

**Published:** 2018-09-24

**Authors:** Tom R. Robinson, Nicholas J. Rosser, Alexander L. Densmore, Katie J. Oven, Surya N. Shrestha, Ramesh Guragain

**Affiliations:** ^a^Department of Geography, Durham University, Durham DH1 3LE, United Kingdom;; ^b^National Society of Earthquake Technology–Nepal, Kathmandu, Nepal

**Keywords:** scenario ensembles, seismic risk, contingency planning, earthquakes, hazard and risk

## Abstract

High death tolls from recent earthquakes show that seismic risk remains high globally. While there has been much focus on seismic hazard, large uncertainties associated with exposure and vulnerability have led to more limited analyses of the potential impacts of future earthquakes. We argue that as both exposure and vulnerability are reducible factors of risk, assessing their importance and variability allows for prioritization of the most effective disaster risk-reduction (DRR) actions. We address this through earthquake ensemble modeling, using the example of Nepal. We model fatalities from 90 different scenario earthquakes and establish whether impacts are specific to certain scenario earthquakes or occur irrespective of the scenario. Our results show that for most districts in Nepal impacts are not specific to the particular characteristics of a single earthquake, and that total modeled impacts are skewed toward the minimum estimate. These results suggest that planning for the worst-case scenario in Nepal may place an unnecessarily large burden on the limited resources available for DRR. We also show that the most at-risk districts are predominantly in rural western Nepal, with ∼9.5 million Nepalis inhabiting districts with higher seismic risk than Kathmandu. Our proposed approach provides a holistic consideration of seismic risk for informing contingency planning and allows the relative importance of the reducible components of risk (exposure and vulnerability) to be estimated, highlighting factors that can be targeted most effectively. We propose this approach for informing contingency planning, especially in locations where information on the likelihood of future earthquakes is inadequate.

## Significance

High death tolls from recent earthquakes have highlighted the need to better identify ways to effectively reduce seismic risk. We address this need by developing a new earthquake scenario ensemble approach. We model impacts from multiple different earthquake scenarios, identifying impacts that are common to multiple scenarios. This method allows us to estimate whether particular impacts are specific to certain earthquakes or occur irrespective of the location or magnitude of the next earthquake. Our method provides contingency planners with critical information on the likelihood, and probable scale, of impacts in future earthquakes, especially in situations where robust information on the likelihood of future earthquakes is incomplete, allowing disaster risk-reduction efforts to focus on minimizing such effects and reducing seismic risk.

Despite global efforts to reduce seismic risk, earthquakes remain one of the deadliest natural hazards worldwide (1). Much of the scientific interest in reducing seismic risk, which is a function of hazard, exposure, and vulnerability, has focused on better understanding of seismic hazard, with a particular focus on refining estimates of recurrence times and probabilities of exceeding given levels of ground motion (2, 3). While hazard assessment is a prerequisite for calculating risk, available data on exposure and functions that model fragility often introduce significant uncertainties. Furthermore, full risk calculations require a holistic analysis of losses, including fatalities, injuries, and financial, infrastructure, property, and indirect losses, so deriving absolute risk is often intractable. Consequently, while there have been several notable advances in the computation of earthquake risk and probable loss at national and global levels (4–10), these have tended to focus on data-rich regions, such as California (11). Despite these efforts, the high death tolls in many recent large earthquakes demonstrate that earthquake risk remains high globally, and in data-poor regions such as the Himalaya may even be increasing as growth in population exposure and vulnerability outpaces the rate of improvement in understanding of seismic hazard (1, 11, 12).

The two most common approaches to seismic hazard analysis (SHA) are probabilistic (PSHA) or deterministic (DSHA). PSHA is a widely used method that identifies all known possible earthquakes that may affect a given site and characterizes their estimated recurrence intervals (13, 14). The resulting output is an estimate of the likelihood of exceeding some value of ground motion at a given location over a given period of time (e.g., a 2% chance of exceedance in 50 y). This is especially useful for determining appropriate seismic design codes for built infrastructure, allowing engineers to establish the maximum strength of shaking that buildings are expected to witness during their design life (14). Despite its sound basis, PSHA can be misunderstood, leading to implementations that attract criticism (15). This is especially true in regions where past earthquake data are sparse (2, 11, 16–18), where spurious probabilities can be generated (11). These criticisms have proved controversial, however (19, 20), and several have been largely rejected (21). Nevertheless, in regions with limited information on future earthquake probabilities different applications of PSHA can result in widely differing hazard and risk estimates, such as recent efforts in Nepal (22).

DSHA focuses on the use of scenarios of individual or small numbers of earthquakes, typically considering either the maximum credible event or the worst-case event that could occur on known active or potentially active faults (14, 23). Shaking from the resulting scenario(s) is derived from attenuation relationships using different likelihoods of exceedance (14). The resulting output shows the strength and extent of shaking expected from the maximum credible or worst-case earthquake with a given likelihood of exceedance, providing an upper limit for planning. This approach also has notable limitations, however, such as (*i*) a focus on one or a small number of events, (*ii*) difficulty in accurately determining the maximum credible event, and (*iii*) a weak statistical basis for estimates of uncertainty (19, 20, 24).

Irrespective of the approach used, the outputs of both are arguably not tailored for contingency planning, where defining risk in terms of the potential consequences of the next future earthquake is the priority concern. Contingency planning operates on two levels: first through planning for times of disaster and second for disaster risk reduction (DRR) (25, 26). Effective planning requires both estimation of the likelihood and scale of future earthquake impacts and understanding of those that are specific to a single earthquake scenario or that could occur in many different earthquakes. Likewise, effective contingency planning requires that we can determine the locations where impacts are most likely to occur, along with the average and worst-case impacts for all locations, so that both emergency relief and preevent DRR activities can be prioritized. Thus, for those tasked with managing earthquake risk, moving beyond probabilities of shaking to probabilities of consequences of future earthquakes is essential (25, 27).

Addressing such complex questions about future events resonates with the challenges faced by climate and meteorological modelers attempting to generate future climate and weather scenarios. They address this through the use of ensembles of models, which consist of suites of scenarios of future climate or weather events based on different conditions and model realizations (28–34). The outputs from all scenarios are then aggregated to identify common elements that are more likely to be realistic representations of future events. Here, we propose a similar approach for the assessment of seismic risk, to derive greater clarity on the potential impacts of future earthquakes. We establish an ensemble of earthquake scenarios, with each individual scenario containing empirically derived estimates of the associated impacts. We then average and compare consequences from all scenarios in the ensemble to examine the emergent impacts, focusing on those that are common to multiple scenarios. Our approach is not intended to supersede either PSHA or DSHA, as no individual analysis is suitable for all intended tasks (14). Instead, we propose the approach as a complementary tool for the assessment of seismic risk with the specific aim of informing earthquake contingency planning. We concentrate here on providing the median and maximum impact estimates, the number of impact-inducing scenarios, the specificity of impacts to individual scenarios, and exceedance probabilities for impacts. We demonstrate the approach using the case of earthquake-induced fatalities in Nepal. Earthquake hazard in Nepal is relatively poorly constrained, leading to often widely differing hazard maps (22), but is thought to be among the highest globally (35–38). Population exposure and vulnerability to earthquakes is also high (39, 40), and previous earthquake impacts have been substantial (41–44), yet impact estimates for future earthquakes are limited (42). While we focus on fatalities, other forms of loss (injuries and financial losses) could also be explored in this manner.

## Materials and Methods

### Method Overview.

**Fig. 1.**
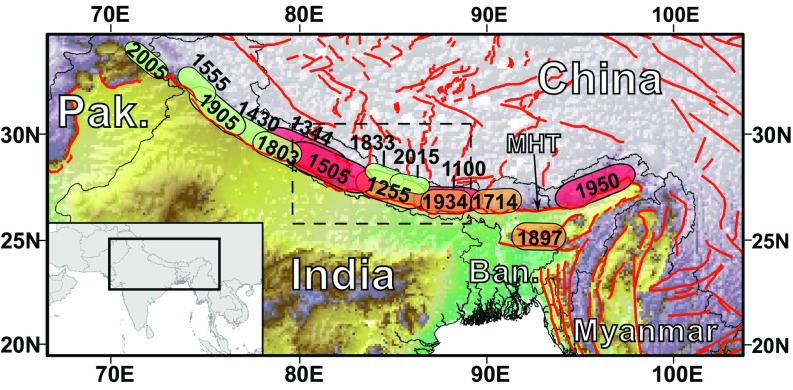
Earthquake history of the Himalayan arc. Numerous large (M_w_ >7.0) earthquakes have been recorded along the MHT system over the last 1,000 y, with little evidence that the largest ruptures are confined to any specific segment. Polygons show known or inferred rupture extents with associated calendar dates and colors represent magnitudes (green, M_w_ 7.0–8.0; orange, M_w_ 8.0–8.5; red, M_w_ 8.5+). Dashed box shows location of Nepal. Red lines show active faults from Taylor and Yin (82). Ban, Bangladesh; Pak, Pakistan.

We adapt the ensemble approach used in climate and meteorological modeling for the purposes of estimating the consequences of future earthquakes. We model the losses associated with 30 different earthquakes that are large enough to cause substantial damage in Nepal at three different times of day to give 90 scenarios, based on our current understanding of active fault locations and potential future earthquakes. The sample of scenarios is chosen based on current understanding of historic earthquakes (Fig. 1) and fault slip rates to give a suite of geologically diverse prototypical scenarios and is large enough that the statistical properties of the results can give some useful insight into the possible consequences of these earthquakes. While each of the modeled earthquakes is plausible, the exact probability of each remains unknown. Instead, each scenario is assigned a uniform probability and weighting in the ensemble. While this approach avoids issues associated with selection of weights based on poorly constrained recurrence intervals, it has important consequences for our results. First, a uniform weighting precludes the ability to discuss “absolute risk,” because the hazard calculations do not include absolute probabilities. Thus, we focus on “relative risk” between scenario outcomes, which we argue is invaluable for earthquake contingency planning. Uniform probabilities will also overemphasize the contribution from the largest-magnitude events, as well as those on upper-plate faults. Conversely and importantly, uniform weighting allows a focus on the role of exposure and vulnerability in producing risk and impacts. This is crucial for contingency planning and DRR, because while earthquake hazard is irreducible, both exposure and vulnerability to earthquakes can be reduced.

While the recurrence interval for each of our scenarios is unlikely to be uniform, previous work has suggested that earthquakes of all magnitudes on the Main Himalayan Thrust (MHT) have ∼500-y recurrence intervals, and that major [moment magnitude (M_w_) >7] earthquakes can be followed by great (M_w_ >8) earthquakes in the same location sooner than plate convergence rates would suggest possible (36). Such observations may explain the relatively short intervals between the 1833 (M_w_ 7.8), 1934 (M_w_ 8.2), and 2015 (M_w_ 7.8) earthquakes in central and eastern Nepal (Fig. 1). Others, however, have suggested that recurrence intervals for the largest magnitude events on the MHT may be on the order of 1,000 y (45). Importantly, however, this highlights that at present we remain unable to assign meaningful recurrence intervals beyond uniform.

In each scenario, we combine estimates of ground shaking with population and building exposure data taken from the most recently available (2011) national population census of Nepal (46) and use previously published, empirical building fragility curves to estimate resulting impacts. We calculate fatalities by Village Development Committee (VDC), which was the third-level administrative division in Nepal up to 2017 and is consistent with the 2011 census data. We then aggregate fatalities and fatality statistics (frequency, median, maximum, and specificity) across the 75 districts and five development regions, which comprised the pre-2017 first- and second-level administrative divisions. We focus on fatalities as a single measure of impact, but other loss measures such as injuries, financial losses, or property losses could equally be calculated. Because we focus on relative risk, the numbers of fatalities discussed below are only indicative of expected impacts, and they are not intended as absolute estimates of likely fatalities in Nepal. Finally, to provide information tailored to earthquake response planners, we also consider social vulnerability, which has been shown to be a significant predictor of earthquake impacts and losses (47). We combine our physical vulnerability metrics with two examples of social vulnerability metrics employed as proxies for disaster vulnerability: the Human Development Index (HDI) (48) and a remoteness index that reflects the need for and ease of providing logistical assistance, to estimate total relative seismic risk for each district of Nepal.

### Modeled Earthquake Scenarios (Hazard).

We choose an ensemble of 30 large (M_w_ >7) earthquake scenarios based on historical records and paleoseismic evidence (Fig. 1), assuming that previously documented earthquakes are representative of potential future earthquakes at decadal-to-centennial time scales (49). Note that this approach cannot account for unanticipated events such as fault linkage or simultaneous rupture of multiple faults (e.g., ref. 38). For known or inferred active faults without historical evidence of earthquakes, geologic data on long-term slip rates and displacement styles, along with fault dimensions and empirical scaling relationships (50), were used to estimate plausible scenario earthquakes.

**Fig. 2.**
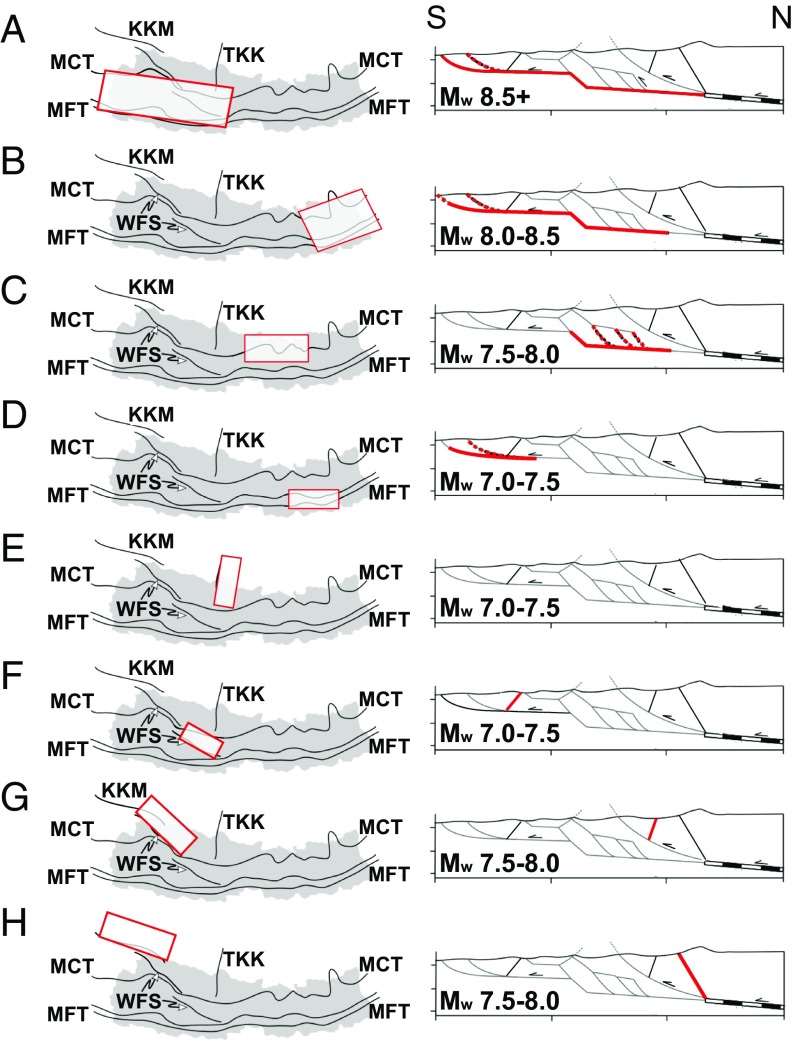
Map and simplified north–south cross-sectional views of the eight prototypical scenario earthquakes in our ensemble. Red-outlined boxes (*Left*) show the surface projection of the assumed failure planes. Thick red lines in (*Right*) show the down-dip extents of fault rupture, while dashed lines show possible simultaneous/alternative rupture scenarios. (*A*) giant (M_w_ 8.5+) earthquakes on the MHT such as the 1505 western Nepal event; (*B*) great (M_w_ 8.0–8.5) earthquakes on the MHT such as the 1934 Nepal–Bihar event; (*C* and *D*) M_w_ 7.0–8.0 ruptures of the lower or upper ramp of the MHT, similar to the 2015 Gorkha event; (*E*) M_w_ 7.0–7.5 ruptures of normal faults in southern Tibet, such as those bounding the Thakkhola graben (note that rupture is not shown in cross-section); (*F*) M_w_ 7.0–7.5 ruptures of the southern portion of the WFS; (*G*) M_w_ 7.5–8.0 ruptures of the northern portion of the WFS; (*H*) M_w_ 7.5–8.0 ruptures of the Karakorum Fault. KKM, Karakorum Fault; MCT, Main Central Thrust; MFT, Main Frontal Thrust; TKK, Thakkhola graben. Data from ref. 51.

In the last 1,000 y, at least 15 M_w_ >7.5 earthquakes have been recorded along the Himalayan arc (Fig. 1) (36–38, 44, 51). The majority of these are associated with the MHT; however, spatial variations in rheology and geometry can limit rupture extent, giving rise to various prototypical forms of MHT earthquake (36, 51). These include (*i*) giant ruptures, such as the 1950 Assam and 1505 western Nepal earthquakes, that initiate near the brittle–ductile transition and rupture to the surface, have lengths >200 km, and have M_w_ >8.5 (36, 37); (*ii*) great ruptures, such as the 1934 Nepal–Bihar earthquake, that are similar to giant ruptures but do not necessarily reach the surface and have M_w_ 8.0–8.5 (35, 44); and partial ruptures, like the 2015 Gorkha event, that rupture either the (*iii*) lower or (*iv*) upper ramp of the MHT and have M_w_ 7.0–8.0, with larger magnitudes anticipated on the lower ramp (52) (Fig. 2). Paleoseismic evidence of great-to-giant earthquakes on the MHT in *ca*. 1100, 1255, and 1344 (Fig. 1) suggests that earthquakes on this fault are not constrained to individual segments within Nepal and can occur on any section of the MHT throughout the Himalayan arc (36, 44, 53).

As well as the MHT, numerous other faults within or close to Nepal have previously sustained, or are capable of sustaining, M_w_ 7+ earthquakes. The largest is the Karakorum Fault, which hosted a M_w_ ∼7.5 earthquake in 1895 (54) and is capable of M_w_ 8.0 events (55). In western Nepal, a set of faults known as the Western Fault System (WFS) partition motion between the MHT and the Karakorum Fault. Quaternary offsets associated with these faults suggest repeated earthquakes since the last glacial advance (56) with evidence of possibly two M_w_ 7+ earthquakes between AD 1165 and 1400 (57). Extension in the southern Tibetan Plateau is accommodated on a series of north–south-striking normal faults, of which the largest, most active, and closest to Nepal are the faults bounding the Thakkhola graben. These have historically sustained M_w_ 6.2–6.4 earthquakes but are likely capable of M_w_ 7+ events (58, 59).

**Fig. 3.**
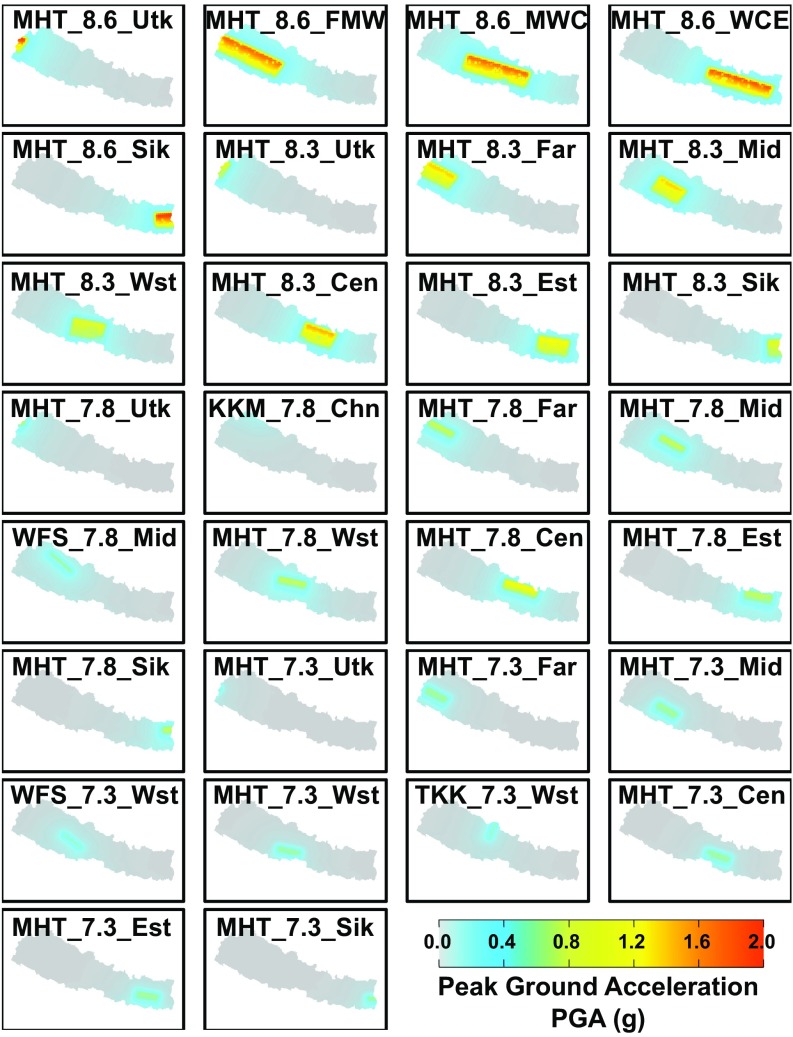
Earthquake scenario ensemble. Modeled ground shaking in terms of PGA with 50% probability of exceedance for the 30 scenario earthquakes in the ensemble. Note that shaking values are only shown for locations within Nepal. Scenario codes are given in the format fault_magnitude_location. Cen, Central Region; Chn, China; Est, East Region; Far, Far-West Region; FMW, Far-West, Mid-West, and West Regions; KKM, Karakorum Fault; Mid, Mid-West Region; MWC, Mid-West, West, and Central Regions; Sik, Sikkim (northeast India); TKK, Thakkhola graben; Utk, Uttarakhand (northwest India); WCE, West, Central, and East Regions.

We therefore consider eight different prototypical scenarios for M_w_ 7+ earthquakes in Nepal (Fig. 2). Earthquakes on upper-plate faults such as the WFS and the Thakkola graben are restricted in their location, whereas those occurring on the MHT are allowed to occur at multiple locations along strike. For the MHT, we assign earthquake magnitudes at the center of the published ranges, comprising (*i*) giant earthquakes with M_w_ 8.6, (*ii*) great earthquakes with M_w_ 8.3, (*iii*) blind lower-ramp earthquakes with M_w_ 7.8, and (*iv*) upper-ramp earthquakes with M_w_ 7.3. We model these earthquakes as occurring between Uttarakhand on Nepal’s western border and Sikkim to the east, incrementally shifting each rupture patch to produce adjacent scenarios that span and extend beyond Nepal to avoid edge effects. In total, we consider five giant scenario earthquakes and seven of each of the great, upper ramp, and blind lower ramp scenario earthquakes (Fig. 3). For the upper-plate faults, we consider events at the upper end of the likely magnitude range: (*v*) a M_w_ 7.8 earthquake on the southern part of the Karakorum Fault, (*vi*) a M_w_ 7.8 event on the northern part of the WFS, (*vii*) a M_w_ 7.3 event on the southern part of the WFS, and (*viii*) a M_w_ 7.3 earthquake in the Thakkhola graben (Fig. 3).

We model the shaking from each of these events in terms of peak ground acceleration (PGA, in units of meters per second^2^) with OpenSHA (60), using the ground motion prediction equations of Abrahamson and Silva (61), exceedance probabilities of 50%, and shallow shear wave velocity (Vs30) derived from topographic slope (62, 63).

### Exposure.

**Fig. 4.**
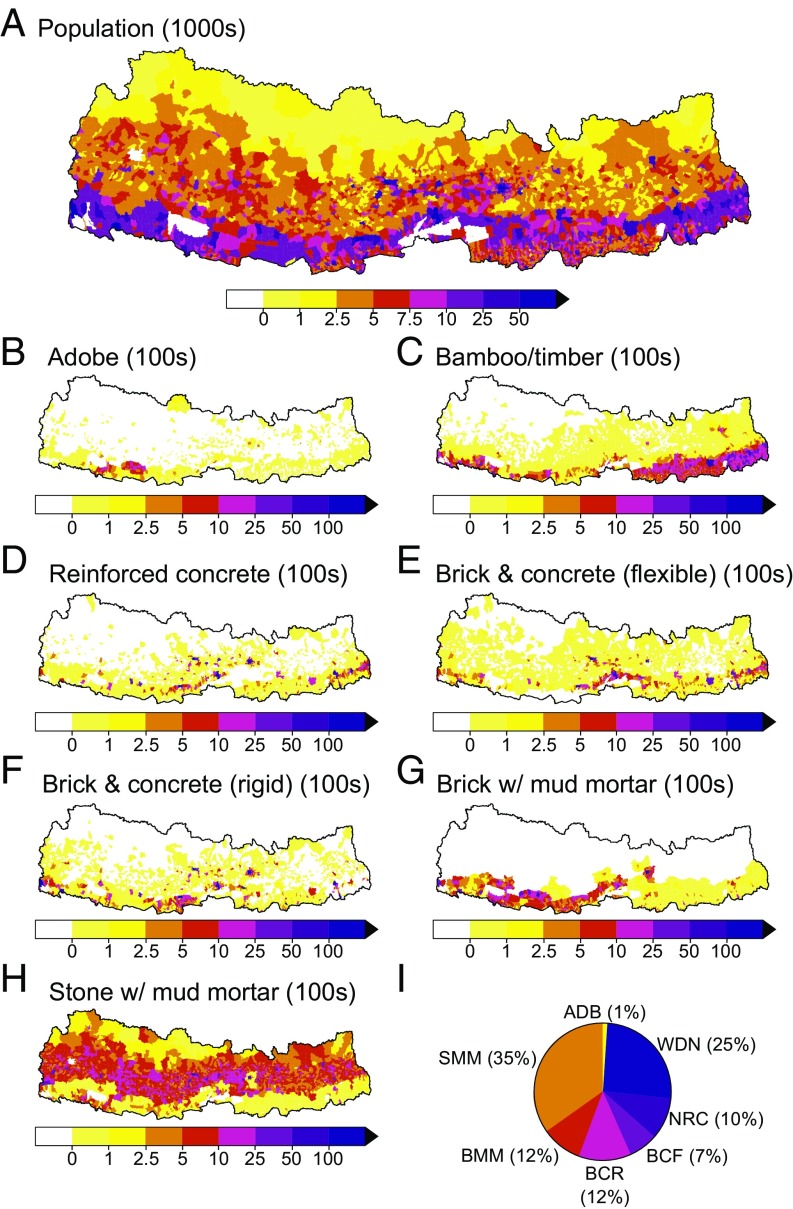
Population and building exposure in Nepal. Total population and number of residential buildings by construction type within each VDC in Nepal according to the National Population and Housing Census (2011). (*A*) population; (*B*) adobe buildings; (*C*) bamboo/timber buildings; (*D*) nonengineered reinforced concrete buildings; (*E*) brick and concrete (flexible flooring) buildings; (*F*) brick and concrete (rigid flooring) buildings; (*G*) brick with mud mortar buildings; (*H*) stone with mud mortar buildings; and (*I*) pie chart showing the percentage of each building type. ADB, adobe; BCF, brick and concrete (flexible flooring); BCR, brick and concrete (rigid flooring); BMM, brick with mud mortar; NRC, nonengineered reinforced concrete; SMM, stone with mud mortar; WDN, bamboo/timber.

We use the National Population and Housing Census 2011 for Nepal to assess the exposure of population and buildings (Fig. 4) to each scenario in the ensemble at the VDC level, the smallest pre-2017 administrative division for which data are available. In the absence of alternative more reliable data, we do not disaggregate by gender or age. The census contains the number of residential buildings per VDC with different types of foundation, roof, and wall construction. Using this information, we classify residential buildings into seven different generic typologies: (*i*) adobe, (*ii*) bamboo/timber, (*iii*) brick and concrete (flexible flooring), (*iv*) brick and concrete (rigid flooring), (*v*) nonengineered reinforced concrete, (*vi*) brick with mud mortar, and (*vii*) stone with mud mortar (Fig. 4). We estimate individual building occupancy by assuming a uniform distribution of people. Shaking exposure for each scenario is derived using the mean modeled PGA per VDC.

While exposure as a function of both daily and seasonal variations in building occupancy is still poorly understood, we account for some temporal differences by deriving building occupancy rates for three different earthquake occurrence times: (*i*) night, (*ii*) day (working), and (*iii*) day (nonworking). We distinguish between urban and rural VDCs by assuming that urban locations have higher occupancy on working days than rural locations, and vice versa. Building occupancy rates (Table 1) are derived in consultation with international humanitarian partners based in Nepal and are subject to a first-order calibration through retrospective fatality modeling of the 2015 Gorkha earthquake (*SI Appendix*). We note, however, that these assumptions and associated uncertainties can be large and so represent a considerable gap in current knowledge.

**Table 1.** Building occupancy rates

	Building occupancy	
Time of day	Urban, %	Rural, %
Night	99	99
Day (working)	70	40
Day (nonworking)	40	70

Assumed residential building occupancy rates for urban and rural VDCs for three different times of day.

### Vulnerability.

We derive total fatality estimates for each scenario by considering the vulnerability of each building typology to seismic shaking, combining locally (64) and globally derived (10, 65) building fragility data where necessary. Based on the work of the Global Earthquake Model–Earthquake Consequence Database (GEM-ECD) (65), we assume that shaking-derived fatalities are limited to collapsed buildings, which correspond to a subsection of the “Complete Damage” state described in HAZUS (10). We therefore calculate the number of buildings suffering complete damage using the relevant fragility curves, before estimating the proportion that collapse based on probabilities from the GEM-ECD (Table 2).

**Table 2.** Building collapse and fatality rates

Building type	Collapse probability, %	Fatality rate, %
Adobe	15.0	5.0
Bamboo/timber	3.0	0.5
Brick and concrete (flexible)	15.0	5.0
Brick and concrete (rigid)	15.0	15.0
Nonengineered reinforced concrete	13.0	10.0
Brick with mud mortar	15.0	5.0
Stone with mud mortar	15.0	5.0

**Fig. 5.**
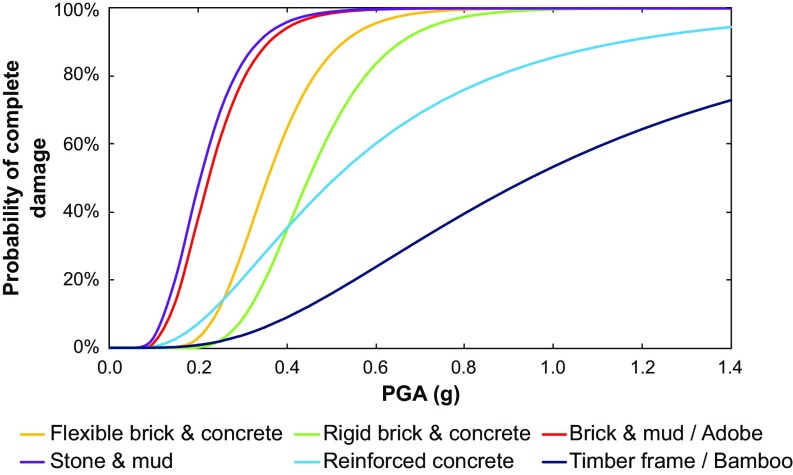
Residential building vulnerability. Empirically derived fragility curves for complete damage (i.e., the structure has collapsed or is in imminent danger of collapse) for different residential building types in Nepal from Guragain (64) and HAZUS (10). Curves for nonengineered reinforced concrete and bamboo/timber buildings correspond to building types C3M (concrete frame with unreinforced masonry infill, midrise, low code) and W1 (wood, light frame, low code) in HAZUS (10), respectively.

Collapse probabilities and fatality rates for different building types in Nepal derived from global empirical relationships and taken from GEM-ECD (65). Collapse probabilities apply only to buildings suffering “complete damage” as defined by HAZUS (10) and calculated from the respective fragility curves (Fig. 5).

For adobe, brick and concrete (flexible flooring), brick and concrete (rigid flooring), brick with mud mortar, and stone with mud mortar buildings, we use available Nepal-specific fragility curves (Fig. 5) from Guragain (64). These predominantly masonry buildings are most prevalent throughout Nepal, accounting for 65% of the total and almost all buildings in rural regions (Fig. 4). For nonengineered reinforced concrete and bamboo/timber buildings, no Nepal-specific fragility curves are available and thus we rely on fragility curves from HAZUS (10), using the curves corresponding to building types C3M (concrete frame with unreinforced masonry infill, midrise, low code) and W1 (wood, light frame, low code), respectively (Fig. 5). We note that these curves were initially developed for the United States and may not be applicable to Nepal. Despite this, the curve for reinforced concrete structures suggests a worse performance than found in recent empirical analysis of building performance during the 2015 earthquake (66) and so is likely to be conservative.

Finally, to estimate total seismic risk by district we combine fatality statistics from the ensemble with two social vulnerability measures: remoteness and human development. Remoteness is a semiquantitative measure of accessibility for each VDC developed by the US Agency for International Development and scored out of 10 (1 = most accessible; 10 = least accessible). It includes factors such as the distance to roads, available transportation methods, and distance from key services. We use remoteness scores (67), averaged across all VDCs in a district and weighted by population, as a measure of predisaster accessibility. In the context of contingency planning, this measure is used as a proxy for the likely scale and speed of postdisaster aid delivery, and by inference, an indicator of high levels of compounded postdisaster vulnerability. It can also be considered as a measure of the likely need for postdisaster assistance, as remote rural communities have been shown to be more likely to require assistance than more accessible urban communities (48). HDI is a summary measure of life expectancy, education, and standard of living, among other factors, and is scored out of 1, where 1 is most developed and 0 is least developed. We use the 2014 HDI scores for each district of Nepal (68) as a proxy for human vulnerability to earthquakes, with lower scoring districts considered more vulnerable. HDI has previously been investigated as an indicator for disaster risk, with higher HDI scores generally associated with lower average losses (48, 69). While both remoteness and HDI have some direct relevance to social vulnerability, these measures are indicative rather than definitive and are not intended to exhaustively capture all dimensions of social vulnerability to disasters. A more definitive discussion of social vulnerability to natural hazards specific to Nepal is provided by Gautam (40).

## Results

### Planning for Disaster.

**Fig. 6.**
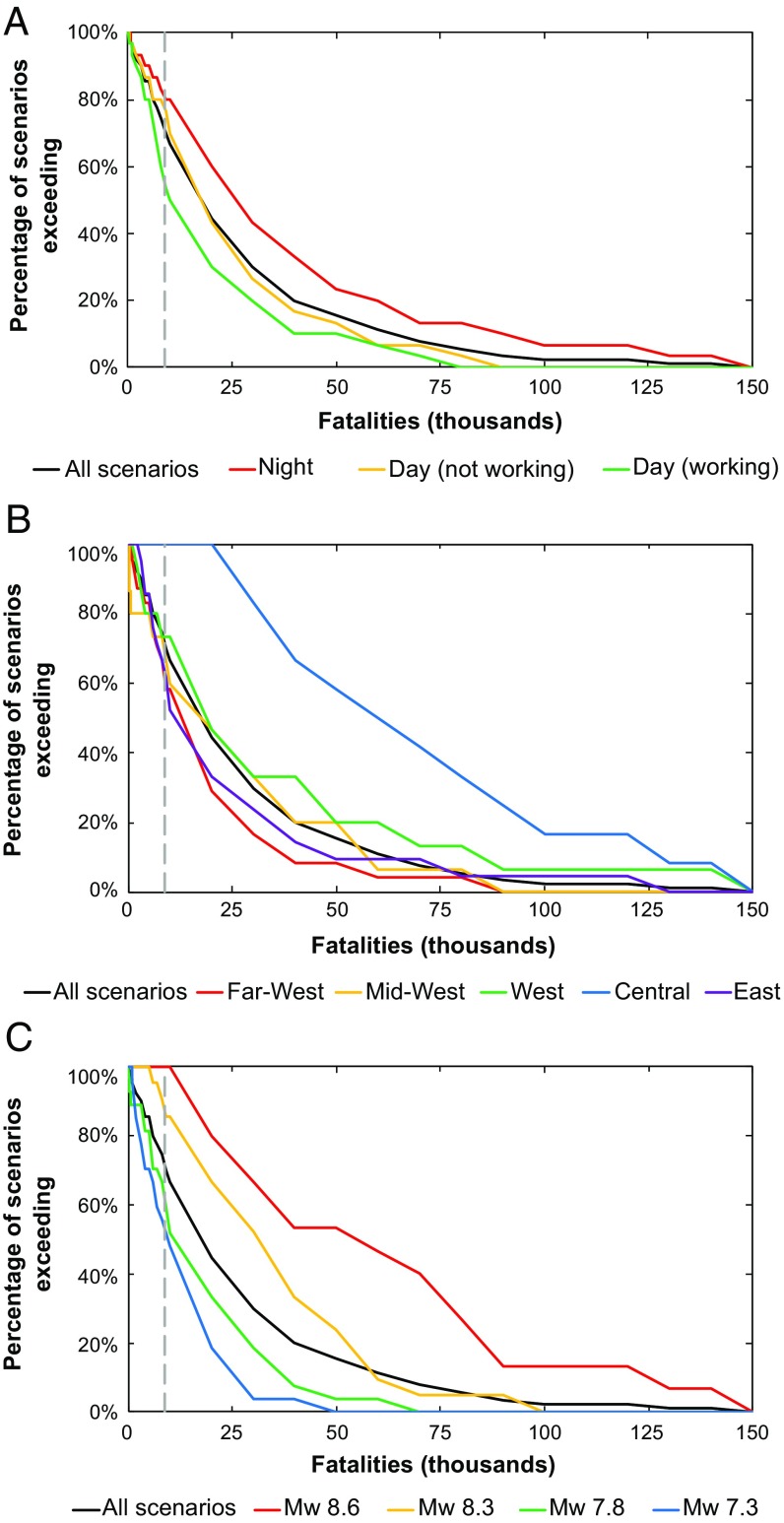
Exceedance probabilities for fatalities. Probabilities are derived from the frequency distribution of scenarios in the entire ensemble compared with different scenario subsets: (*A*) time of day, (*B*) location of scenario earthquake (based on pre-2017 development regions), and (*C*) earthquake magnitude. The dashed gray line shows the number of fatalities recorded in the 2015 Gorkha earthquake (70).

Because the exact nature of the next earthquake to occur is unknowable, we use our ensemble to estimate the relative scale of fatalities in the next earthquake, irrespective of its nature, by assessing the frequency distribution of total earthquake fatalities for all scenarios (Fig. 6). We find that over 70% of modeled scenarios result in more than the ∼9,000 fatalities experienced in the 2015 Gorkha earthquake (70), while 16% exceed ∼50,000 fatalities, and 2% exceed ∼100,000 fatalities. Based on our assumptions about building occupancy rates, there is a substantial increase in risk for nighttime compared with daytime earthquakes. At night, 50% of scenarios exceed ∼23,000 fatalities and 5% exceed ∼125,000 fatalities, compared with ∼10,000 fatalities and ∼65,000 fatalities, respectively, for daytime earthquakes (Fig. 6). Earthquakes in the Central Region incur the greatest losses, with 50% of scenarios exceeding ∼60,000 fatalities and 5% exceeding ∼144,000 fatalities, compared with ∼11,000 fatalities and ∼54,000 fatalities, respectively, for earthquakes in the Far-West Region. Only the M_w_ 8.6 scenarios generate in excess of ∼100,000 fatalities, while no M_w_ 7.3 scenario results in >50,000 fatalities.

### Risk Metrics.

#### Fatality exceedance probabilities.

**Fig. 7.**
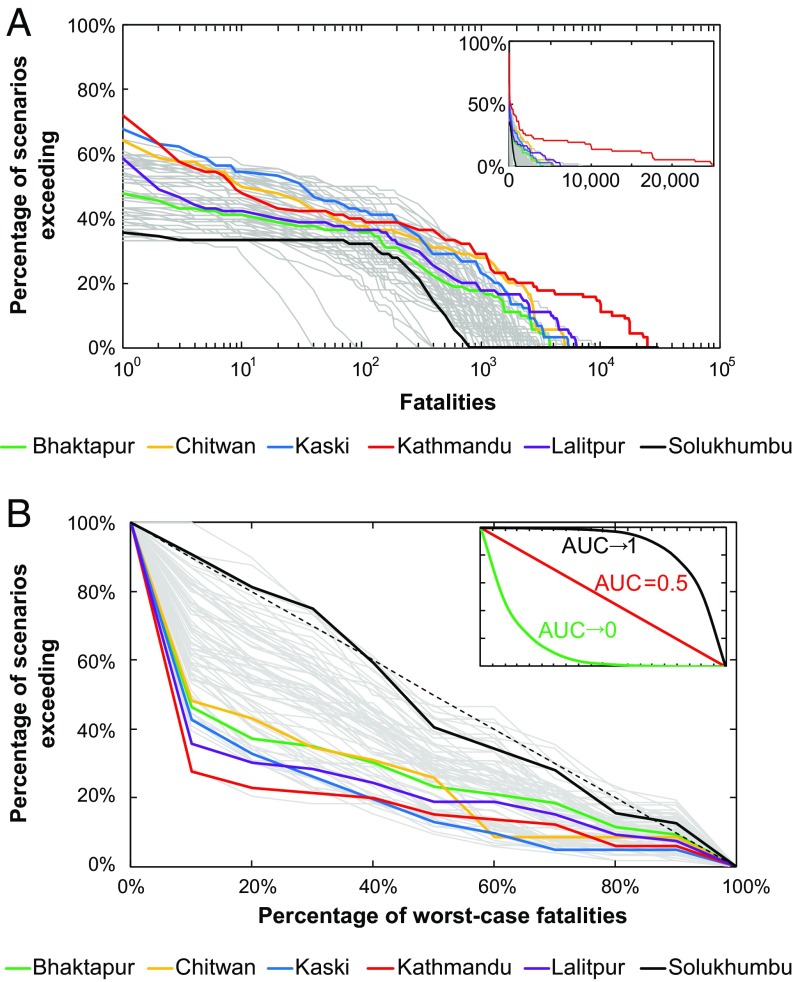
Fatality exceedance probability (*A*) and impact specificity (*B*) for all 75 districts of Nepal. (*A*) Percentage of scenarios in the ensemble with fatalities exceeding given values for each district. *Inset* shows same data on a linear scale. (*B*) Specificity of impacts in terms of their variability based on all scenarios causing >0 fatalities, normalized with respect to the worst-case scenario for each district. (*Inset*) Schematic definition of the data: Lines with convex-up curvature (black) show that the majority of impacts are close to the maximum (area under the curve, AUC, approaching 1), while lines with concave-up curvature (green) show that the majority of impacts are close to the minimum (AUC approaching 0). Both represent low specificity as impacts show little variability with different scenarios. Linear distributions (red) show that impacts are evenly distributed (AUC ∼ 0.5) and thus represent high specificity. In both panels, six key districts are highlighted: Kathmandu (red), Bhaktapur (green), and Lalitpur (purple) comprise the Kathmandu Valley and Nepal’s largest urban area; Kaski (blue) and Chitwan (yellow) host two of Nepal’s other largest cities (Pokhara and Bharatpur, respectively) and are popular tourist destinations; Solukhumbu (black) is home to Mt. Everest and the Everest Base Camp trek, which is one of the most popular treks in Nepal. All other districts are shown in gray.

**Fig. 8.**
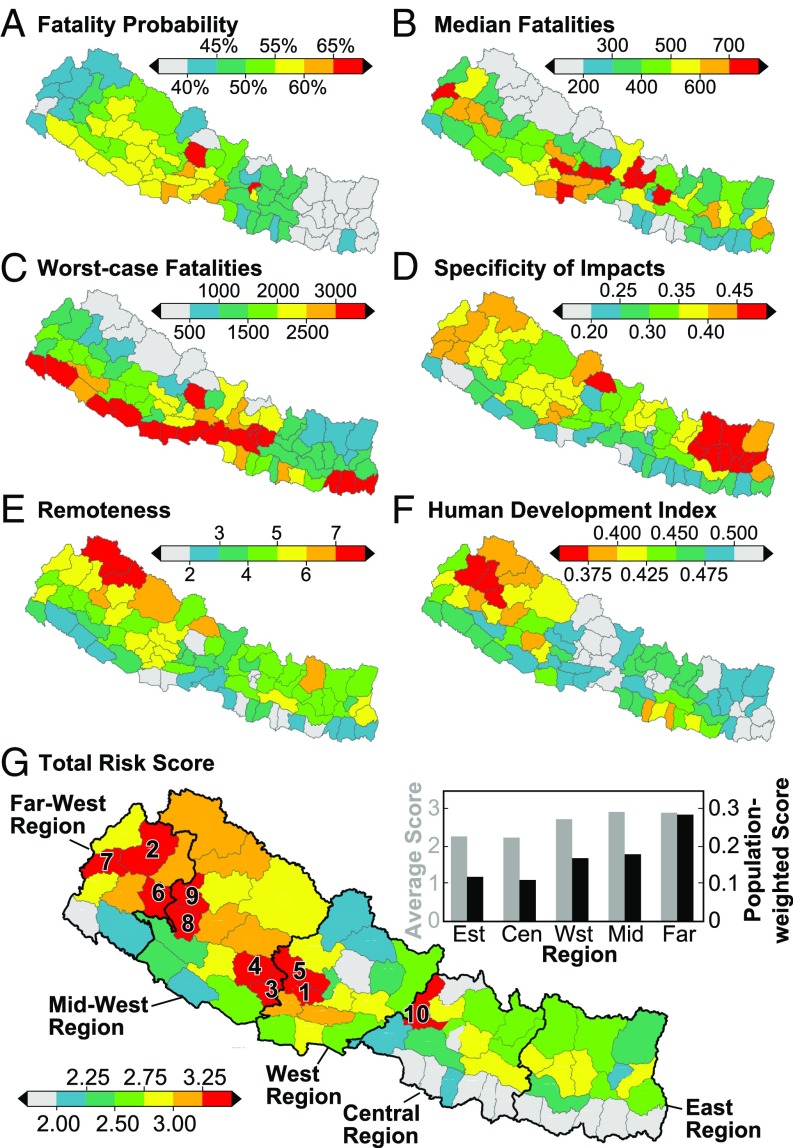
Seismic risk for Nepal. Spatial distribution of relative seismic risk in Nepal based on summary statistics for modeled fatalities from the ensemble combined with two social vulnerability metrics: (*A*) percentage of scenarios with at least one fatality, (*B*) median fatalities for all scenarios that cause fatalities, (*C*) maximum fatalities, (*D*) specificity of fatalities for all scenarios that cause fatalities, (*E*) remoteness score, (*F*) HDI, and (*G*) total relative seismic risk, calculated as the normalized sum of all six risk metrics. Numbers in *G* show district ranks.

We estimate the relative scale of fatalities by district from the frequency distribution output from the entire ensemble (Fig. 7). A total of 72% of scenarios result in fatalities in Kathmandu, the largest percentage of fatal scenarios for any district (Figs. 7 and 8). Districts in the East Region have the fewest number of fatal scenarios, typically <40% (Figs. 7 and 8). While this may appear to be an edge effect, the impacts of scenarios occurring across the eastern border in Sikkim were included in the ensemble, and a similar result is not seen in the Far-West Region related to the high proportion of timber/bamboo buildings (Fig. 4). Importantly, as all districts have one or more fatalities in at least one-third of the scenarios, seismic risk is high for the whole country.

#### Median and worst-case fatalities.

Median modeled fatalities are highest in Kavrepalanchok and the majority of the largest modeled fatality totals are in the West and Central Regions (Fig. 8). Generally, districts that border China have the lowest median fatalities, although notably some heavily populated districts in the south also have low median fatalities. In Gorkha, Dhading, Lalitpur, and Nuwakot, the median fatalities are equivalent to those experienced in the 2015 earthquake, suggesting that, in this sense, the 2015 earthquake was a “typical” event in these districts.

Maximum fatality estimates broadly correlate with the population distribution (Fig. 4), with the three Kathmandu Valley districts (Kathmandu, Lalitpur, and Bhaktapur) and the majority of districts in the south having the largest worst-case fatalities (Figs. 7 and 8). Kathmandu has the largest worst-case fatalities at >24,000. Notably, in Rasuwa and Sindhupalchok the maximum modeled fatalities are equivalent to those recorded in 2015, suggesting that the Gorkha earthquake was close to a worst case for those districts.

### Specificity of Impacts.

Understanding how the impacts might vary under different earthquake scenarios is as important to contingency planners as the median and worst-case impacts. If all scenarios in the ensemble result in similar impacts in a district, then the district can be considered to have low hazard specificity. Alternatively, if impacts are highly variable across the ensemble, then a district has high hazard specificity, as the impacts are intrinsic to a precise scenario and so there is more uncertainty about what could happen in the next event. For contingency planning, low specificity is preferable, even when associated with large impacts, as planners can be confident of the scale of impacts to be expected. For high-specificity locations, impacts are intimately linked to whichever earthquake occurs, but as this cannot be anticipated, specificity could inform planning decisions.

To calculate specificity, we determine the frequency distribution of impacts by district with respect to the corresponding worst-case scenario. The distribution is used to obtain the percentage of scenarios with fatalities exceeding a given fraction of the worst case (Fig. 7). Calculating the area under the curve (AUC) indicates how losses are skewed toward either the minimum (AUC → 0), worst-case (AUC → 1), or are evenly distributed (AUC ∼ 0.5). (Fig. 7). Specificity is considered to be highest when AUC = 0.5 and reduces as AUC tends to 0 or 1.

All districts have an AUC between 0 and 0.53, showing that impacts are either evenly distributed or skewed toward the minimum. Crucially, no district has impacts skewed toward the worst case (Fig. 7). Worst-case impacts occur in very few scenarios, and the large majority of impacts are far less than the maximum. For example, in Kathmandu 75% of fatality-inducing scenarios result in fatalities that are <15% of the worst case. Importantly, there is large variation in specificity across Nepal: high-specificity districts are mostly clustered in the East Region, while low-specificity districts are along the southern border (Fig. 8). For 55 of the 75 districts in Nepal, at least two-thirds of modeled scenarios result in impacts that are <50% of the worst case (Fig. 7). This suggests that contingency planning for these districts should focus on median losses, as impacts approaching the worst case are likely to be rare. For the remaining districts, planning should focus on the worst-case impacts as fatalities are variable and dependent on the precise scenario that occurs.

### Prioritization for Risk Reduction.

With finite resources available for risk-reduction efforts, contingency planning requires an objective approach to prioritize DRR efforts toward locations that are most at-risk. To help inform this, we estimate the total relative seismic risk for all districts in Nepal by combining the probability of fatalities, the median and maximum fatalities, and the specificity of fatalities with remoteness and HDI. We give each district a normalized score out of 1 for all six risk metrics, such that the district considered most at risk (i.e., with the lowest value of HDI and the highest value for all other metrics) scores 1, and then simply sum for all of the metric scores assuming a uniform weighting. We recognize that others may see value in alternate weightings of the metrics, and so we provide the raw scores in *SI Appendix*, Tables S2 and S3.

Using our combination, we find that total seismic risk is notably higher in western areas of Nepal (Fig. 8). Gulmi in the West Region is the most at-risk district with a score of 3.61, and the nearby districts of Rolpa, Pyuthan, and Baglung account for three of the next four most at-risk districts, demonstrating that this area has the highest seismic risk in Nepal. Saptari in the East Region has the lowest risk, although many districts on the southern border, particularly in the East and Central Regions, have comparable scores.

The middle-to-low score for Kathmandu district (2.61) is particularly notable. While Kathmandu has high scores for the frequency of fatalities and the worst-case scenario, its low specificity, low remoteness, and comparatively high HDI all help to reduce its total relative seismic risk compared with other districts in Nepal. It is striking that while Kathmandu commonly features on global rankings of high-seismic-risk cities, ∼9.5 million Nepalis (∼35% of the total population) live in districts with higher risk scores than the capital, and the 10 most at-risk districts in Nepal contain a total population of ∼2.5 million, comparable to the population of the capital. Applying alternate weightings to each of the risk metrics changes the values and specific ranking of individual districts, but a similar overall pattern of higher risk scores in the west and a middle-to-low score for Kathmandu generally remains (*SI Appendix*, Fig. S3).

## Discussion

The intention of this study is to outline an approach to the assessment of seismic risk that focuses on the importance of the reducible components of risk, namely exposure and vulnerability. We argue that this is critical for identifying and prioritizing the most pressing risk-reduction activities and the most at-risk locations at a national level. We do not intend for what we propose to supersede either PSHA or DSHA, but instead to complement them by specifically addressing the needs of contingency planners. It is therefore important to highlight the limitations of our ensemble approach and possibilities for further research.

First, it is important to consider whether an ensemble can account for the full range of potential future earthquakes. We consider only a small number (8) of prototypical scenario earthquakes, although we allow their locations to vary. It is not clear how our results depend on the number of scenarios that are included in our ensemble, although in future this could be tested. Small changes in earthquake magnitude (∼0.1–0.2) compared with the larger steps between scenarios included here are unlikely to affect our results, because ground motion saturation occurs at M_w_ 7.3–7.5, beyond which point the main factor controlling shaking strength is distance to the fault. Small increases (or decreases) in magnitude are also unlikely to require significant changes in fault dimensions and therefore will not significantly alter the spatial pattern of shaking or its impacts. We do not consider earthquakes smaller than M_w_ ∼7.0 because their impacts are likely to be smaller than what are typically considered by contingency planners (for example, the 1988 Dharan and 2011 Sikkim earthquakes, both M_w_ 6.9), although they may still cause considerable disruption if they affect a major population center. While there is some evidence that earthquakes larger than M_w_ 8.6, perhaps approaching M_w_ 9.0, are possible along the Himalayan arc (71), this remains contentious (38). Given the scale of potential impacts from M_w_ 8.6 events compared with the extent of Nepal, however, the scale of impacts from an M_w_ 9.0 event may not be substantially larger (*SI Appendix*, Fig. S2). Our scenarios only consider relatively simple fault rupture patterns, ignoring more complex ruptures such as those described by Hamling et al. (72); however, incorporating such complexity into our model requires more advanced seismic modeling, which is beyond the scope of this study. The potential amplification of ground motion by sedimentary basins, such as the Kathmandu Valley, is also an important factor that has not been included in this study, along with secondary hazards and cascading hazards such as landsliding and liquefaction. We note, however, that recent improvements in coseismic landslide modeling, including our ongoing work on this topic (73–75), allow some of these effects to be incorporated into future more holistic iterations of this approach. Given that the effects of coseismic landslides appear to be more pronounced among rural mountainous communities (76), their inclusion may not significantly alter the general pattern of relative seismic risk established here.

Alternatives to the assignment of uniform weights to all scenarios in the ensemble may also require further exploration. Herein, we have used a uniform weighting because of gaps in our understanding of earthquake recurrence along the Himalayan arc, and thus the likelihood of each scenario earthquake in our ensemble is unknown. The suggestion that earthquakes of all magnitudes on the MHT may have similar recurrence times (36) may in part support this assumption. However, while this may be true for the MHT, it is unlikely to be so for ruptures of the other upper-plate faults included in our ensemble. In locations where recurrence intervals are better constrained, or where Gutenberg–Richter relationships are well known, these could be used to derive appropriate nonuniform weightings for use in the ensemble.

A further limitation relates to assumptions made around short- and long-term population exposure, where basic research could significantly improve the accuracy of our results. Distributing the population equally between each building type is likely to be an unrealistic proxy for exposure. Key differences in occupancy are known between building typologies: reinforced concrete buildings in Nepal are typically multistory and able to house several families, whereas wooden and adobe buildings are smaller and usually only house a single family. The collapse of the former building typology therefore likely underestimates impacts, while collapse of other building types may overestimate impacts.

Assumptions around the population exposure at different times of day are also poorly constrained. Our initial assumptions are based on discussions with humanitarian agencies in Nepal but are likely to be a gross oversimplification. In reality, the difference in population exposure between working and nonworking days, particularly in rural areas, may be less pronounced than assumed here. Further, the population exposure is likely to be highly spatially variable and not well represented by simple definitions of urban and rural VDCs. We presently lack sufficient information to fully investigate the effect of temporal variations in exposure; while a simple analysis of night versus day has been undertaken, a more nuanced analysis is required to investigate how exposure varies diurnally, particularly around communal times such as meals, and also through the seasons. For instance, we would expect that population movements change significantly during the monsoon period and during the Tihar and Desai festivals (77), but the effect of these on earthquake risk is yet to be addressed. Although we have attempted to calibrate occupancy rates using the 2015 earthquake (*SI Appendix*, Fig. S1), we note that it is not possible from the available data to determine whether the departure of the model results relates to limitations in the occupancy rates, the shaking estimates, the building fragility curves or, more likely, some combination of these factors.

While assumptions around population exposure play an important role in controlling specific impacts, we highlight that these assumptions have been kept consistent throughout our ensemble. Thus, while the number of fatalities presented is not intended to be absolute, the relative differences between districts should remain unchanged unless there are significant differences in the movements of people within different districts beyond the urban and rural distinction employed. Limitations associated with population exposure serve to further highlight the need for a more holistic approach to seismic risk analyses. Even if it were possible to predict the precise timing and nature of a future earthquake, we remain unable to effectively estimate its impacts if we cannot accurately account for exposure.

## Implications and Conclusions

Advances in our understanding of seismic hazard have long shown that for locations such as the Himalayan arc, it is not a matter of whether a devastating earthquake will occur, but when. It is therefore essential to reduce earthquake risk where possible and to prepare for this eventuality. We presently remain unable to predict the precise timing or nature of future earthquakes, and thus their resulting impacts. To date, the assessment of seismic risk has focused primarily on improving understanding of earthquake hazard in terms of potential ground shaking, which has resulted in major advances (78). Nevertheless, for contingency planning, the precise geophysical nature of the earthquake that next occurs is of lesser importance than its impacts (25–27). Thus, finding an approach that provides insight on what impacts are most likely to happen, and that can complement methods to assess seismic hazard, has obvious benefits.

We present an approach to estimating relative seismic risk that relies on an ensemble of scenarios representing potential future earthquakes. This approach is particularly well-suited to countries like Nepal, where earthquake hazard is relatively poorly understood, information on earthquake recurrence intervals is limited, and earthquake hazard maps contain widely differing results. Our approach weights all plausible future large earthquakes equally, allowing us to focus on elements of vulnerability and exposure that contribute to relative seismic risk. Our work shows that it is possible to assess the range of potential impacts and to consider how specific impacts relate to specific earthquakes. For the majority of districts in Nepal, similar impacts occur irrespective of the scenario earthquake, and these impacts are typically closer to the minimum than the worst case. This suggests first that the scale of impacts expected in a future earthquake can already be relatively well constrained, and second that planning for the worst-case impacts may place an unnecessarily large burden on the limited resources available. Instead, the optimal level of mitigation that minimizes the total cost to society, including both the cost of expected impacts and the cost of mitigation (22, 79, 80), may require planning for losses significantly smaller than the worst case.

Our results also imply that, while Kathmandu is regarded as one of the most seismically at-risk cities in the world (37, 81), greater relative seismic risk exists in the rural western areas, particularly in Gulmi and neighboring districts. This suggests that, while the whole of Nepal requires urgent earthquake risk-reduction activities, rural western districts are in particular need. A sole planning focus on urban earthquake risk in Kathmandu may therefore be inappropriate, as many rural populations within Nepal are at greater relative risk.

## Supplementary Material

Supplementary File
