# Supplementary material for: Use of scenario ensembles for deriving seismic risk
Source: Proc Natl Acad Sci U S A. 2018 Sep 24;115(41):E9532–41. doi: 10.1073/pnas.1807433115 (PMC6187155; doi:10.1073/pnas.1807433115)
Supplement: Supplementary File [file pnas.1807433115.sapp.pdf]

PNAS
www.pnas.org

Supplementary Information for

The use of scenario ensembles for deriving seismic risk

Tom R Robinson, Nicholas J Rosser, Alexander L Densmore, Katie J Oven, Surya N Shrestha, Ramesh Guragain

Tom R Robinson
Email: tom.robinson@durham.ac.uk

This PDF file includes:

- Supplementary text
- Figs. S1 to S3
- Tables S1 to S3
- References for SI reference citations

www.pnas.org/cgi/doi/10.1073/pnas.1807433115

Supplementary Information Text

Fatality model validation

To evaluate the fatality modelling approach, we undertook a two-stage validation process. First, we modelled the impacts resulting from the 2015 Gorkha earthquake based on recorded shaking from the USGS and compared these with observed fatality data provided by the United Nations Office for Coordination of Humanitarian Affairs (1), which were collated from police reports at the time of the earthquake. Secondly, we compared our results with those of other published models, comparing like-for-like scenarios where available.

Comparison to 2015 earthquake. The 2015 Gorkha earthquake occurred on Saturday 25 April 2015 at 11:56 Nepal standard time and killed a total of 8712 people across a total of 39 districts, although 97% of fatalities occurred in just 10 districts (Fig S1). Using the most up-to-date shaking data available from the USGS (2) we modelled the fatalities expected from our model based on the census data, our vulnerability curves, and our assumed exposure rates for a daytime non-working day event. Our model gave an output of 16,933 fatalities spread across 20 districts in total but with 95% of fatalities occurring in 13 districts (Fig S1). The model output therefore overestimated fatalities by a factor of 1.9. Whilst this appears large, the complexities associated with earthquake fatality modelling have led others to argue that any model capable of estimating fatalities to within a factor of 2 is useful (3). Our output is also similar to other models for this event, notably the USGS PAGER system (4), which gave a 34% chance of 1000-10,000 fatalities and a 29% chance of 10,000-100,000 fatalities for the event (2), and the Swiss-based QLARM system, which gave an early estimate of 10,000-50,000 before decreasing its estimate to 800-9300 (3).

Our model is capable of estimating the spatial distribution of losses also, which for our study is arguably more important than the total number of fatalities per scenario in order to support decision making on prioritization. Comparing the spatial distribution of modelled and observed losses suggests that our model is useful. Despite notable errors, the model successfully captures the main spatial extent of losses and broadly identifies the relative impacts between districts (Fig S1).

Comparison to published models. Several other fatality models for earthquake scenarios in Nepal have been previously published and we compared these to corresponding scenarios within our ensemble (Table S1). Using the QLARM system, Wyss (5) estimated fatalities resulting from two M_w 8.1 scenario earthquakes centred in Nepal: one in the Far-West Region and one in the Central Region beneath Kathmandu. Because we do not include a M_w 8.1 scenario, we compare these results to our M_w 7.8 and M_w 8.3 scenarios in the same locations (scenario codes MHT_7.8_Far, MHT_7.8_Cen, MHT_8.3_Far, and MHT_8.3_Cen). It should be noted however, that Wyss (4) did not restrict fatalities to Nepal as we do, instead estimating all fatalities including those that would occur in neighbouring China and/or India. Using fatality rates from the 2015 Gorkha earthquake, Sapkota et al. (6) estimated the potential number of fatalities resulting from a recurrence of the 1934 Bihar-Nepal earthquake in the East Region, which they considered to have M_w 8.2. Here, we compare this to our M_w 8.3 scenario in East Region (MHT_8.3_Est). Sapkota et al. (5) provided fatality estimates for Nepal and India separately allowing us to directly compare our results. Similarly, Dixit et al. (7) estimated the number of fatalities likely to result

from a recurrence of the 1934 earthquake, although their estimates only accounted for fatalities in the Kathmandu Valley. The results of these scenarios compared with our own scenarios are shown in Table S1 and show that our results are broadly consistent with previous efforts.

Potential M_w 9.0 scenario

It has been suggested from estimates of accumulated and released strain that there is potential for an M_w 9.0 earthquake along the Himalayan arc (8). However, this is thought to be contentious (9) and we therefore do not include such a scenario in our ensemble. Nevertheless, the fatalities expected from an M_w 9.0 earthquake are not substantially larger than the total worst-case scenario included in our ensemble (~144,000 fatalities). Here, we model the fatalities resulting from an M_w 9.0 scenario for different times of day, assuming a rupture length that spans the entire east-west length of Nepal (Fig S2). Comparatively, such an event produces just ~20% more total fatalities than the MHT_8.6_MWC event in our ensemble, with ~91,000 for a working day, ~109,000 for a non-working day, and 182,000 for a night-time event (Fig S2). The MHT_8.6_MWC scenario in our scenario resulted in ~74,000 fatalities for a working day, ~85,000 for a non-working day, and 144,000 for a night-time event. Notably, there is no change in the worst-case impacts for individual districts. The increase in total fatalities results from a larger number of districts experiencing fatalities at or close to the previously defined worst-case impacts. Nevertheless, such numbers are particularly concerning considering that our analysis only includes impacts within Nepal, and does not include inevitable impacts in neighbouring China and, particularly, northern India, where > 300 million people live.

District risk metric data

We have compiled data on various risk metrics for each district of Nepal based on our scenario ensemble as well as remoteness (10) and human development index (11). The raw data and corresponding relative risk scores displayed in Fig 8 are detailed in Tables S2 & S3. The effect of three alternative weighting schemes (high remoteness, high worst-case impacts, and high median impacts) on total relative seismic risk are shown in Fig S3. These results show that, while the relative risk score and rank for any single district can change, the overall pattern of higher relative seismic risk in western areas of Nepal is consistent across multiple weighting regimes.

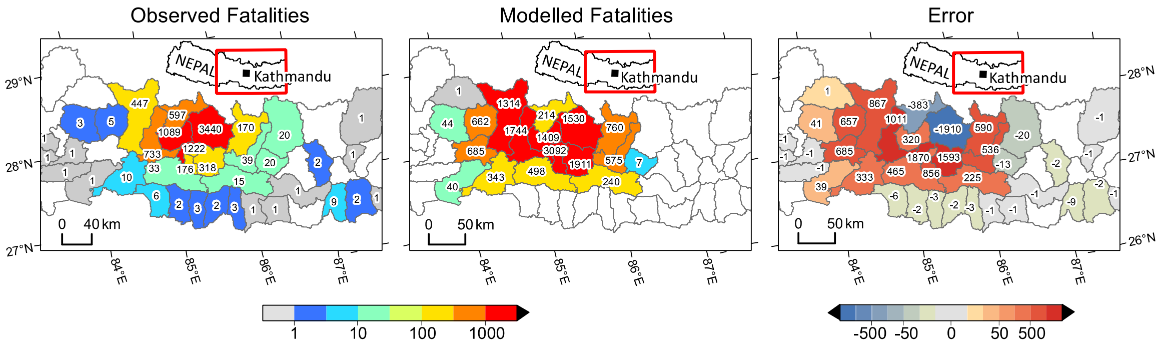

Fig. S1. Comparison of the observed fatalities from the 2015 Gorkha earthquake with modelled fatalities using the census data, vulnerability curves, and exposure rates for a daytime non-working day event.

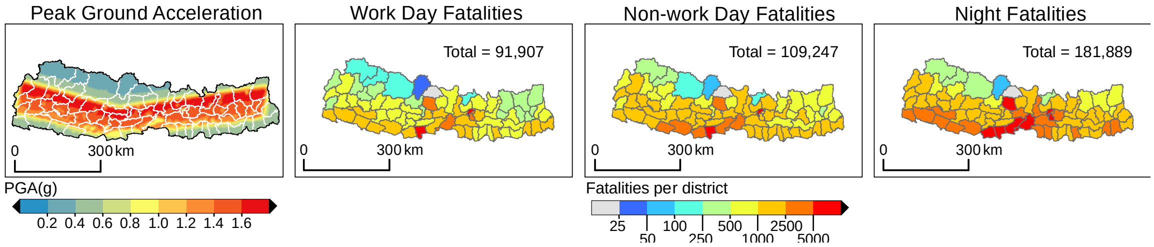

Fig. S2. A M_w 9.0 scenario earthquake for Nepal. Shaking and resulting fatalities for three different exposure times for a potential M_w 9.0 earthquake with a rupture length that covers the entire east-west extent of Nepal.

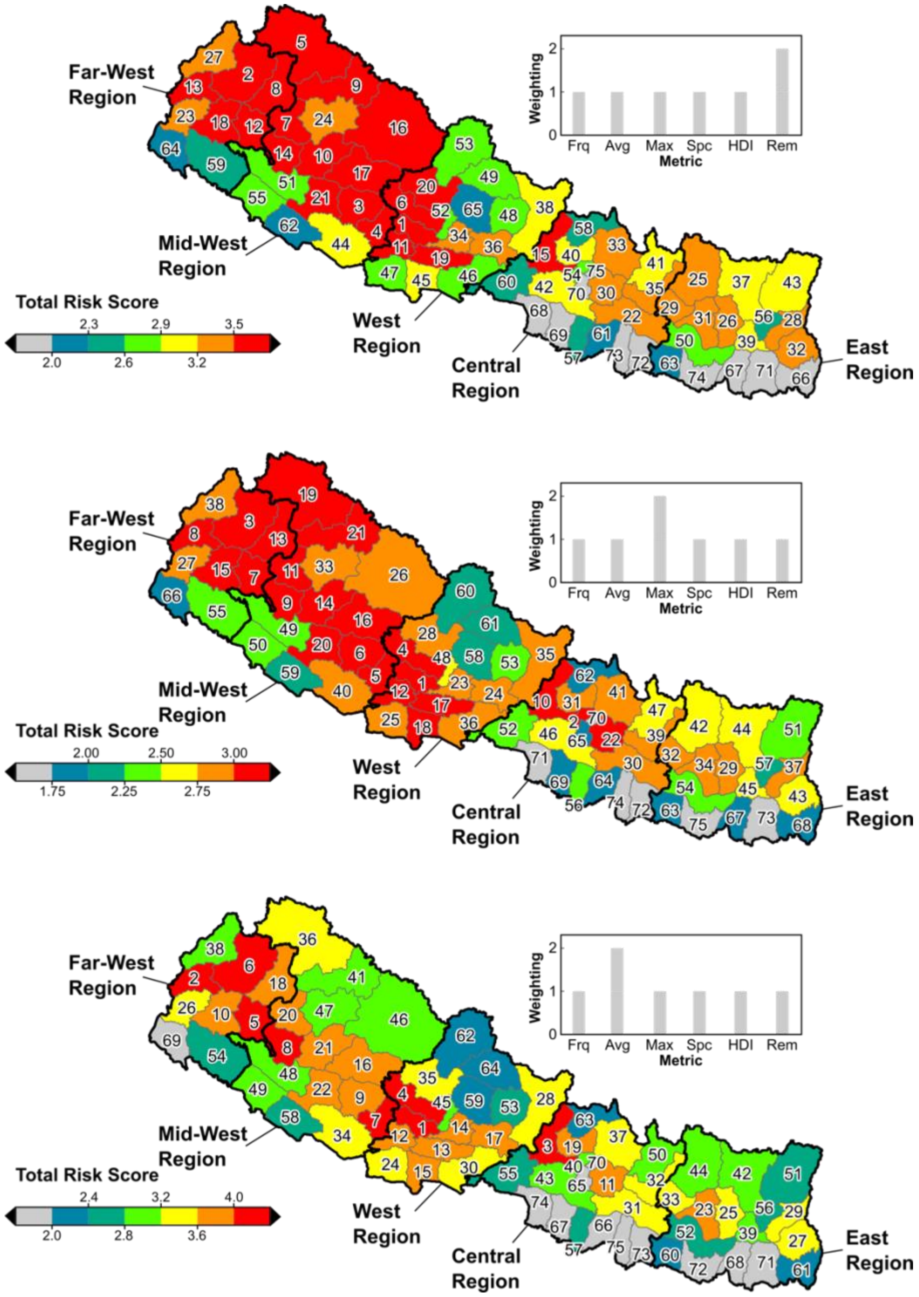

Fig. S3. Total seismic risk for each district of Nepal using three alternative weightings of the risk metrics presented in the text. Despite total risk scores and ranks for individual districts changing with each weighting scheme, the overall pattern of higher seismic risk in western areas of Nepal remains.

Table S1. Comparison between modelled fatalities for similar earthquake scenarios. A summary of published fatality estimates for different earthquake scenarios in Nepal compared with similar scenarios in our ensemble (italics). The range of fatalities quoted for this study accounts for the different times of day modelled.

Scenario magnitude	Scenario Location	Modelled fatalities	Reference
8.1	Far West Region	11,000 – 22,000*	(4)
7.8	*Far West Region*	*8,000 – 20,000*	*This study*
8.3	*Far West Region*	*16,000 – 36,000*	*This study*
8.1	Central Region (Kathmandu)	21,000 – 42,000*	(4)
7.8	*Central Region*	*37,000 – 68,000*	*This study*
8.3	*Central Region*	*53,000 – 98,000*	*This study*
8.2	East Region	33,000	(5)
8.3	East Region	40,000†	(6)
8.3	*East Region*	*24,000 – 59,000*	*This study*

*Includes fatalities in neighboring China and India.
†Only accounts for fatalities within the Kathmandu Valley.

Table S2. Risk metric data. Raw data for each district of Nepal for each of the six risk metrics used to derive relative seismic risk in Fig 8.

District	% scenarios causing fatalities	Median fatalities	Maximum fatalities	Specificity	Remoteness	HDI
Achham	47.78%	682	1910	0.397	4.30	0.378
Argakhachi	55.56%	530	1444	0.400	5.74	0.482
Baglung	58.89%	672	2057	0.384	5.33	0.478
Baitadi	43.33%	796	1916	0.429	4.35	0.416
Bajhang	43.33%	559	1461	0.417	5.76	0.365
Bajura	48.89%	353	988	0.386	5.86	0.364
Banke	56.67%	357	3765	0.209	2.02	0.475
Bara	43.33%	292	2850	0.224	1.85	0.457
Bardiya	56.67%	458	2567	0.262	2.62	0.466
Bhaktapur	47.78%	356	3743	0.283	1.21	0.573
Bhojpur	35.56%	501	1234	0.491	4.87	0.479
Chitwan	64.44%	366	5030	0.267	2.15	0.551
Dadeldhura	40.00%	453	1050	0.425	5.15	0.442
Dailekh	57.78%	603	1955	0.352	4.71	0.422
Dang	57.78%	573	4287	0.256	3.18	0.485
Darchula	43.33%	365	1120	0.376	5.42	0.436
Dhading	47.78%	802	2589	0.369	4.92	0.461
Dhankuta	36.67%	498	1065	0.456	4.72	0.517
Dhanusa	47.78%	145	2832	0.201	1.47	0.431
Dolakha	46.67%	304	1398	0.355	4.65	0.459
Dolpa	54.44%	69	264	0.348	6.80	0.401
Doti	43.33%	636	1573	0.409	4.59	0.407
Gorkha	54.44%	518	2066	0.344	3.80	0.481
Gulmi	53.33%	823	2144	0.423	5.21	0.464
Humla	41.11%	133	342	0.420	7.62	0.376
Ilam	33.33%	609	1422	0.443	5.18	0.526
Jajarkot	57.78%	317	1253	0.348	5.90	0.393
Jhapa	36.67%	436	3006	0.274	2.25	0.518
Jumla	58.89%	109	796	0.337	5.35	0.409
Kailali	57.78%	382	3721	0.196	2.60	0.460
Kalikot	53.33%	321	1010	0.375	6.02	0.374
Kanchanpur	43.33%	227	3084	0.209	3.54	0.475
Kapilbastu	60.00%	638	4847	0.241	1.94	0.432
Kaski	67.78%	341	5406	0.209	1.95	0.576
Kathmandu	72.22%	466	24880	0.196	1.05	0.632
Kavrepalanchok	46.67%	851	3203	0.345	4.01	0.520

Khotang	33.33%	625	1451	0.520	4.37	0.494
Lalitpur	58.89%	300	6229	0.239	1.48	0.601
Lamjung	51.11%	287	1391	0.333	4.46	0.507
Mahottari	41.11%	85	1437	0.239	1.49	0.388
Makwanpur	47.78%	504	3344	0.285	4.51	0.497
Manang	35.56%	16	37	0.491	6.10	0.568
Morang	42.22%	277	3142	0.239	2.29	0.513
Mugu	52.22%	111	397	0.356	7.26	0.397
Mustang	42.22%	31	84	0.424	4.95	0.508
Myagdi	52.22%	326	851	0.395	5.96	0.490
Nawalparasi	60.00%	603	4937	0.248	2.83	0.493
Nuwakot	41.11%	739	2131	0.388	3.22	0.466
Okhaldhunga	38.89%	432	1069	0.470	4.81	0.468
Palpa	57.78%	630	2066	0.340	4.97	0.500
Panchthar	33.33%	504	1254	0.510	5.16	0.498
Parbat	51.11%	440	1164	0.396	3.05	0.510
Parsa	54.44%	78	2480	0.172	2.47	0.464
Pyuthan	56.67%	544	1679	0.379	5.41	0.413
Ramechhap	46.67%	502	1493	0.374	4.27	0.468
Rasuwa	40.00%	94	320	0.358	4.25	0.461
Rautahat	46.67%	230	1772	0.252	3.36	0.386
Rolpa	57.78%	487	1636	0.363	5.49	0.395
Rukum	60.00%	441	1532	0.359	4.80	0.431
Rupandehi	64.44%	769	8919	0.197	1.97	0.498
Salyan	53.33%	496	1814	0.354	4.58	0.441
Sankhuwasabha	36.67%	404	891	0.465	4.85	0.488
Saptari	33.33%	271	1722	0.273	1.47	0.437
Sarlahi	46.67%	122	1760	0.217	3.08	0.402
Sindhuli	47.78%	422	1399	0.343	5.40	0.440
Sindhupalchok	45.56%	465	2229	0.323	4.93	0.455
Siraha	37.78%	302	2035	0.244	2.76	0.408
Solukhumbu	35.56%	321	739	0.488	6.05	0.502
Sunsari	38.89%	294	3103	0.284	2.19	0.496
Surkhet	53.33%	446	2523	0.265	3.64	0.476
Syangja	61.11%	703	2469	0.326	3.43	0.527
Tanahu	56.67%	705	2817	0.325	3.41	0.506
Taplejung	35.56%	336	835	0.444	4.76	0.494
Terathum	34.44%	287	708	0.521	3.76	0.527
Udayapur	34.44%	383	1115	0.395	4.36	0.475

Table S3. Risk metric scores. Raw scores [0-1] for each risk metric for each district of Nepal used to estimate total relative seismic risk in Fig 8.

District	% scenarios causing fatalities	Median fatalities	Maximum fatalities	Specificity	Remoteness	HDI	Risk Score	Rank
Achham	0.312	0.125	0.229	0.211	0.189	0.444	1.509	6
Arghakhanchi	0.114	0.014	0.486	0.578	0.945	0.877	3.014	12
Baglung	0.018	0.002	0.229	0.790	0.593	0.463	2.093	5
Baitadi	0.371	0.033	0.486	0.698	0.746	0.530	2.864	7
Bajhang	0.703	0.197	0.686	0.238	0.271	0.519	2.613	2
Bajura	0.866	0.084	0.200	0.677	0.329	0.619	2.776	11
Banke	0.498	0.042	0.143	0.935	0.571	0.612	2.801	60
Bara	0.735	0.082	0.629	0.528	0.596	0.493	3.061	66
Bardiya	0.584	0.049	0.000	0.998	0.625	0.500	2.756	51
Bhaktapur	0.507	0.045	0.457	0.702	0.304	0.455	2.471	73
Bhojpur	0.074	0.098	0.543	0.000	0.216	0.627	1.558	26
Chitwan	0.632	0.066	0.600	0.650	0.663	0.817	3.429	55
Dadeldhura	0.581	0.059	0.343	0.633	0.490	0.612	2.718	24
Dailekh	0.093	0.011	0.171	0.584	0.486	0.638	1.985	8
Dang	0.256	0.070	0.343	0.251	0.352	0.918	2.189	44
Darchula	0.563	0.064	0.629	0.600	0.676	0.884	3.417	34
Dhading	0.509	0.060	0.686	0.587	0.571	0.750	3.163	10
Dhankuta	0.902	0.358	0.800	0.076	0.140	0.500	2.775	45
Dhanusa	0.574	0.072	0.514	0.571	0.537	0.713	2.981	71
Dolakha	0.465	0.034	0.086	0.920	0.577	0.537	2.620	46
Dolpa	0.305	0.068	0.000	0.317	0.063	0.728	1.481	20
Doti	0.127	0.069	0.343	0.139	0.309	0.858	1.845	14
Gorkha	0.486	0.055	0.371	0.536	0.662	0.716	2.827	35
Gulmi	0.538	0.088	0.314	0.474	0.590	0.660	2.664	1
Humla	0.342	0.080	0.114	0.225	0.259	0.836	1.857	16
Ilam	0.365	0.028	0.057	0.990	0.761	0.485	2.686	40
Jajarkot	0.333	0.123	0.143	0.351	0.174	0.507	1.632	13
Jhapa	0.514	0.100	0.514	0.290	0.394	0.582	2.394	68
Jumla	0.823	0.098	0.714	0.484	0.361	0.392	2.872	28
Kailali	0.825	0.112	0.600	0.478	0.359	0.470	2.844	56
Kalikot	0.383	0.032	0.057	0.853	0.564	0.515	2.404	9
Kanchanpur	0.325	0.027	0.029	0.964	0.413	0.392	2.148	65
Kapilbastu	0.440	0.043	0.029	0.700	0.504	0.586	2.301	36
Kaski	0.312	0.125	0.229	0.211	0.189	0.444	1.509	62
Kathmandu	0.114	0.014	0.486	0.578	0.945	0.877	3.014	42
Kavre	0.018	0.002	0.229	0.790	0.593	0.463	2.093	21
Khotang	0.371	0.033	0.486	0.698	0.746	0.530	2.864	32

Lalitpur	0.703	0.197	0.686	0.238	0.271	0.519	2.613	67
Lamjung	0.866	0.084	0.200	0.677	0.329	0.619	2.776	52
Mahottari	0.498	0.042	0.143	0.935	0.571	0.612	2.801	72
Makwanpur	0.735	0.082	0.629	0.528	0.596	0.493	3.061	47
Manang	0.584	0.049	0.000	0.998	0.625	0.500	2.756	59
Morang	0.507	0.045	0.457	0.702	0.304	0.455	2.471	74
Mugu	0.074	0.098	0.543	0.000	0.216	0.627	1.558	18
Mustang	0.632	0.066	0.600	0.650	0.663	0.817	3.429	58
Myagdi	0.581	0.059	0.343	0.633	0.490	0.612	2.718	23
Nawalparasi	0.093	0.011	0.171	0.584	0.486	0.638	1.985	43
Nuwakot	0.256	0.070	0.343	0.251	0.352	0.918	2.189	30
Okhaldhunga	0.563	0.064	0.629	0.600	0.676	0.884	3.417	29
Palpa	0.509	0.060	0.686	0.587	0.571	0.750	3.163	17
Panchthar	0.902	0.358	0.800	0.076	0.140	0.500	2.775	33
Parbat	0.574	0.072	0.514	0.571	0.537	0.713	2.981	48
Parsa	0.465	0.034	0.086	0.920	0.577	0.537	2.620	70
Pyuthan	0.305	0.068	0.000	0.317	0.063	0.728	1.481	3
Ramechhap	0.127	0.069	0.343	0.139	0.309	0.858	1.845	37
Rasuwa	0.486	0.055	0.371	0.536	0.662	0.716	2.827	61
Rautahat	0.538	0.088	0.314	0.474	0.590	0.660	2.664	54
Rolpa	0.342	0.080	0.114	0.225	0.259	0.836	1.857	4
Rukum	0.365	0.028	0.057	0.990	0.761	0.485	2.686	15
Rupandehi	0.333	0.123	0.143	0.351	0.174	0.507	1.632	31
Salyan	0.514	0.100	0.514	0.290	0.394	0.582	2.394	19
Sankhuwasabha	0.823	0.098	0.714	0.484	0.361	0.392	2.872	41
Saptari	0.825	0.112	0.600	0.478	0.359	0.470	2.844	75
Sarlahi	0.383	0.032	0.057	0.853	0.564	0.515	2.404	64
Sindhuli	0.325	0.027	0.029	0.964	0.413	0.392	2.148	27
Sindhupalchok	0.440	0.043	0.029	0.700	0.504	0.586	2.301	39
Siraha	0.312	0.125	0.229	0.211	0.189	0.444	1.509	63
Solukhumbu	0.114	0.014	0.486	0.578	0.945	0.877	3.014	38
Sunsari	0.018	0.002	0.229	0.790	0.593	0.463	2.093	69
Surkhet	0.371	0.033	0.486	0.698	0.746	0.530	2.864	50
Syangja	0.703	0.197	0.686	0.238	0.271	0.519	2.613	22
Tanahu	0.866	0.084	0.200	0.677	0.329	0.619	2.776	25
Taplejung	0.498	0.042	0.143	0.935	0.571	0.612	2.801	49
Tehrathum	0.735	0.082	0.629	0.528	0.596	0.493	3.061	57
Udayapur	0.584	0.049	0.000	0.998	0.625	0.500	2.756	53

References

1. United Nations Office for the Coordination of Humanitarian Affairs (2015) Nepal Earthquake Facts and Figures. Available at: https://www.undispatch.com/nepal-earthquake-facts-and-figures/ [Accessed March 5, 2018].
2. United States Geological Survey (2017) M 7.8 – 36km E of Khudi, Nepal. Available at: https://earthquake.usgs.gov/earthquakes/eventpage/us20002926#shakemap [Accessed April 26, 2018].
3. Wyss M (2017) Four loss estimates for the Gorkha M7.8 earthquake, April 25, 2015, before and after it occurred. Nat Hazards 86(S1):141–150.
4. Jaiswal K, Wald D, Earle PS, Porter KA, Hearne M (2011) Earthquake casualty models within the USGS Prompt Assessment of Global Earthquakes for Response (PAGER) system. In Spence R (Ed) *Human Casualties in Earthquakes*, 29.
5. Wyss M (2005) Human losses expected in Himalayan earthquakes. Nat Hazards 34(3):305–314.
6. Sapkota SN, Bollinger L, Perrier F (2016) Fatality rates of the M_w ~8.2, 1934, Bihar–Nepal earthquake and comparison with the April 2015 Gorkha earthquake. Earth, Planets Sp 68(1):40.
7. Dixit AM, Ryuichi Y, Dahal RK, Prakash NB (2013) Initiatives for earthquake disaster risk management in the Kathmandu Valley. Nat Hazards 69:631–654.
8. Stevens VL, Avouac J-P (2016) Millenary $M_w > 9.0$ earthquakes required by geodetic strain in the Himalaya. Geophys Res Lett 43(3):1118–1123.
9. Bilham R (2005) Apparent Himalayan slip deficit from the summation of seismic moments for Himalayan earthquakes, 1500 – 2000. Curr Sci 88(10):1658–1663.
10. USAID-Nepal (2016) Quantifying remoteness. Available at: http://aiddata.org/blog/quantifying-remoteness-a-scale-of-accessibility-across-nepal [Accessed December 11, 2017].
11. United Nations Development Programme (2014) Nepal Human Development Report 2014: Beyond Geography Unlocking Human Potential.
